# Image sharpening improves the visibility of reference points on low-dose lateral cephalometric radiographs: a phantom study

**DOI:** 10.1093/dmfr/twag047

**Published:** 2026-07-06

**Authors:** Ikuho Kojima, Arata Ito, Yusuke Shimada, Naoko Tagaino Watanabe, Manami Ikawa, Shu Onodera, Hiroyasu Kanetaka, Masahiro Iikubo

**Affiliations:** Department of Dental Informatics and Radiology, Tohoku University Graduate School of Dentistry, Sendai, Miyagi 980-8575, Japan; Department of Medical AI Center, Tohoku University Hospital, Sendai, Miyagi 980-8574, Japan; Department of Orthodontics and Dentofacial Orthopedics, Tohoku University Graduate School of Dentistry, Sendai, Miyagi 980-8575, Japan; Department of Dental Informatics and Radiology, Tohoku University Graduate School of Dentistry, Sendai, Miyagi 980-8575, Japan; Department of Dental Informatics and Radiology, Tohoku University Graduate School of Dentistry, Sendai, Miyagi 980-8575, Japan; Department of Radiological Technology, Tohoku University Hospital, Sendai, Miyagi 980-8574, Japan; Department of Radiological Technology, Tohoku University Hospital, Sendai, Miyagi 980-8574, Japan; Department of Orthodontics and Dentofacial Orthopedics, Tohoku University Graduate School of Dentistry, Sendai, Miyagi 980-8575, Japan; Department of Dental Informatics and Radiology, Tohoku University Graduate School of Dentistry, Sendai, Miyagi 980-8575, Japan; Department of Medical AI Center, Tohoku University Hospital, Sendai, Miyagi 980-8574, Japan

**Keywords:** image processing, cephalometric radiography, radiation dose reduction

## Abstract

This phantom study aimed to determine whether a novel non-artificial intelligence image-sharpening approach improves the visibility of reference points on low-dose lateral cephalometric radiographs. Lateral cephalometric radiographs were obtained using an adult human bone phantom at 100% of the routine dose. Low-dose imaging was performed using 60%, 40%, and 20% of the routine dose. Each low-dose image underwent a 2-level image-sharpening process using the Medical Image Enhancer software. Six reviewers evaluated the visibility of the 5 reference points in the first and second sets of sharpened and 100% radiation-dose images. Using Scheffé’s method of paired comparisons, odds ratios (ORs) were reported as relative values for a standard cephalometric radiograph (100% radiation dose) to compare the visibility of reference points among imaging conditions. For the first set of mildly sharpened 60%-dose images, the ORs for the 4 reference points were >1.0, Point A (OR 1.12, *P* = .83), Nasion (OR 2.26, *P* = .11), posterior nasal spine (OR 2.35, *P* = .1), and incisal edge of the maxillary central incisor (OR 1.35, *P* = .53), indicating visibility comparable to or better than the 100%-dose images. For the second set of markedly sharpened images, rated visibility was significantly higher for all reference points at 60% and 40% doses and for several reference points at 20% dose. Image-sharpening algorithms improved the visibility of reference points on low-dose phantom radiographs, suggesting their technical feasibility as a post-acquisition approach for dose-reduced cephalometric imaging.

## Introduction

Maxillofacial radiographic examinations are widely used as diagnostic tools in pediatric dentistry to help achieve treatment goals. Unlike other diagnostic tools, there are potential risks associated with the use of ionizing radiation; therefore, the benefits must outweigh the risks. Deterministic effects are not of concern regarding diagnostic radiation; however, stochastic effects cannot be completely ignored. Pediatric populations are more susceptible to ionizing radiation.^1^ This increased susceptibility arises from multiple factors. Underaged patients present with higher mitotic activity, which inherently leads to higher radiation sensitivity in tissues that are less developed and contain more undifferentiated cells.^2^ They also have a longer life expectancy, which means a protracted period to receive background radiation, and a prolonged period in which cancer can develop. It is essential to implement concrete measures for the “optimization of protection” in order to keep radiation doses as low as reasonably achievable.^3^

New techniques have been developed to improve medical images by using image-sharpening algorithms that are not arbitrary. Recently developed non-artificial intelligence (AI) image-sharpening algorithms, designed to enhance fine structural details, can markedly improve visibility. These methods have been deployed across diverse domains, including night-time infrared surveillance and underwater imaging, and are currently being translated into medical imaging. These algorithms have been used in ophthalmology to assess radiographic and magnetic resonance images,^4^ and photographs of the fundus of the eye in ophthalmology.^5–8^ These studies have demonstrated that image-sharpening algorithms can improve the clarity of the surgical field. Proprietary image-sharpening algorithms enhance image quality by optimizing the contrast and restoring the resolution of images by narrowing the point-spread function. This is achieved by reducing the skewness and kurtosis of the light distribution of the objects of interest.

This technical report describes the application of an image-sharpening algorithm to low-dose cephalometric radiographs and presents a preliminary phantom-based visibility assessment. The primary purpose of cephalometric imaging is to characterize maxillofacial skeletal morphology. By identifying reference points and performing cephalometric analysis (eg, construction of facial diagrams), lateral cephalometric radiographs are routinely used to diagnose malocclusion and plan orthodontic treatments. We focused on cephalometry among the plain radiographic modalities because these examinations are frequently performed in young patients. If image sharpening improves the visibility of reference points on dose-reduced images, this approach may represent a useful technique for further investigation in low-dose cephalometric radiography. This phantom study aimed to evaluate whether image sharpening improves the visibility of reference points on low-dose lateral cephalometric radiographs.

## Methods

### Imaging technique

An adult dry human skull embedded in an acrylic resin phantom (Sanwa Kaseihin, Osaka, Japan) was positioned in a patient-like orientation using the ear rod of the head fixator for lateral cephalometric radiography. Dose measurements and phantom radiographs were obtained using a cephalometric radiographic unit (CX-150 W, ASAHI Roentgen IND, Kyoto, Japan) employing a storage-phosphor plate and image reader system (Fuji IP cassette type C and FCR Speedia; FUJIFILM Medical Co. Ltd., Tokyo, Japan). The subject-focus distance was 200 cm. The subject-imaging plate cassette distance was 20 cm. The routine protocol was as follows: 80 kVp, 200 mA, and 100 ms (the dose area product [DAP]: 270 mGy cm^2^) (Figure 1A). Low-dose acquisitions at 60%, 40%, 20%, and 10% of the routine dose were obtained by shortening the exposure time to 60 ms (DAP: 162 mGy cm^2^), 40 ms (108 mGy cm^2^), 20 ms (54 mGy cm^2^), and 10 ms (27 mGy cm^2^), respectively, while maintaining 80 kVp and 200 mA constant (Figure 1B-E).

**Figure 1:**
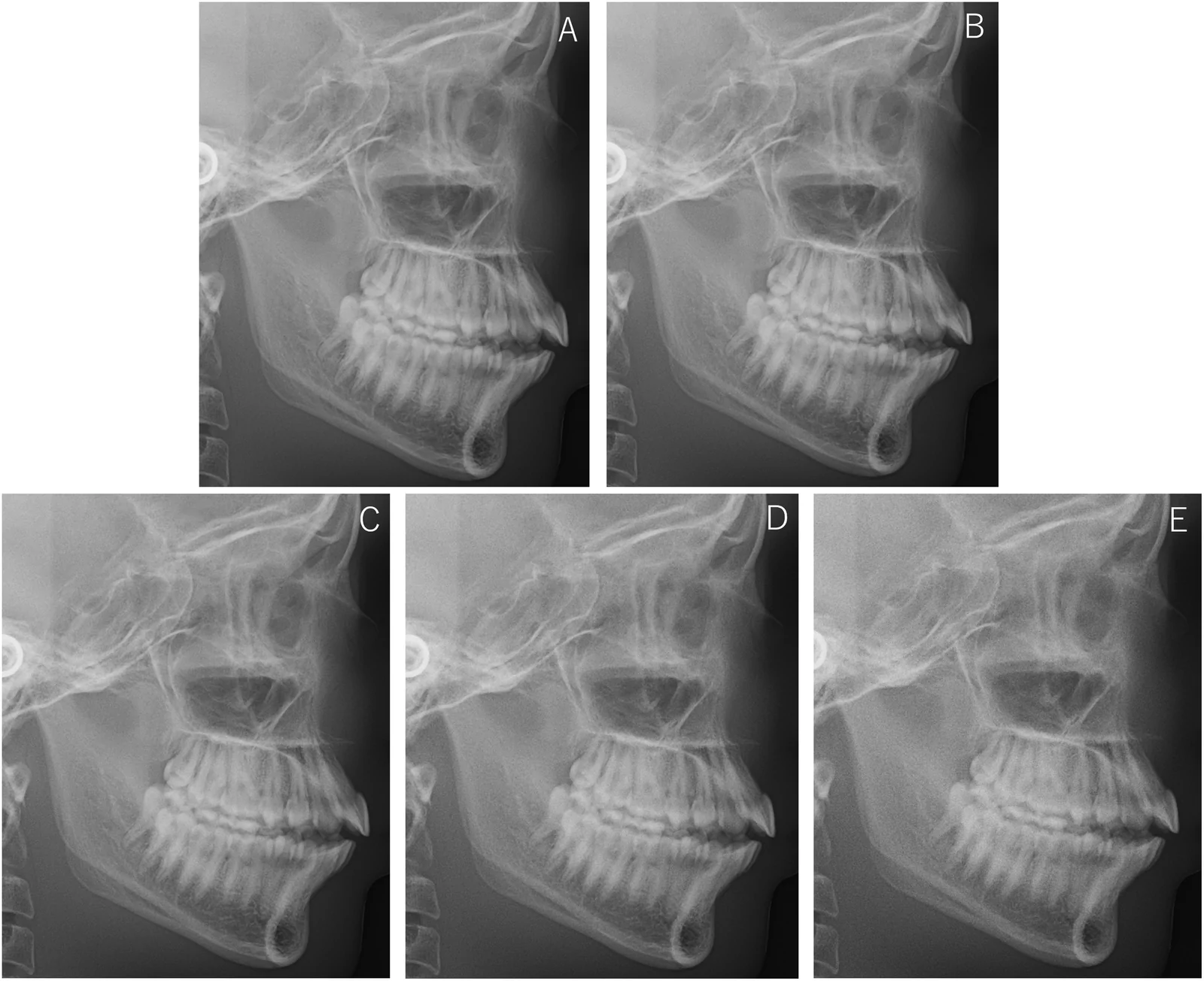
Cephalometric radiographs of 100% radiation dose and low dose (60%, 40%, 20%, and 10%). (A) Routine clinical cephalometric radiograph acquired at a 100% radiation dose. (B-E) Low-dose cephalometric radiograph acquired at 60%, 40%, 20%, and 10% radiation doses, respectively.

### Image processing

The cephalometric radiographs were processed using image-sharpening software (Medical Image Enhancer: MIEr; Logic & Design Inc., Tokyo, Japan). Subsequently, the image was sharpened by applying contour enhancement only in a specific frequency region according to the fiber diameter, while simultaneously suppressing the restoration of the removed comb structure. The image-sharpening process was also used to improve the visibility in low-contrast areas. The algorithm used in this study can perform real-time image processing; however, it was not implemented in the study.

Three low-dose cephalometric radiographs acquired at 60%, 40%, and 20% of the routine clinical radiation dose were processed with 2 levels of image sharpening. The mildly sharpened image used a sharpening correction strength of 55% and a contrast correction ratio of 20% (Figure 2A-C). The markedly sharpened image used a sharpening correction strength of 70% and a contrast correction ratio of 70% (Figure 2D-F). For the mild-sharpened images, a board-certified oral and maxillofacial radiologist adjusted all processing parameters, including sharpening, to enhance sharpness while ensuring that the pre- and post-processed images would be indistinguishable at first glance. Processing was applied cautiously so that subsequent reviewers could not immediately recognize that post-processing had been performed. For the marked sharpening of images, the same oral and maxillofacial radiologist adjusted the parameters to maximize the visibility of the cephalometric reference points, even if this yielded an overall appearance that would make the post-processing apparent at first glance to the subsequent reviewers.

**Figure 2:**
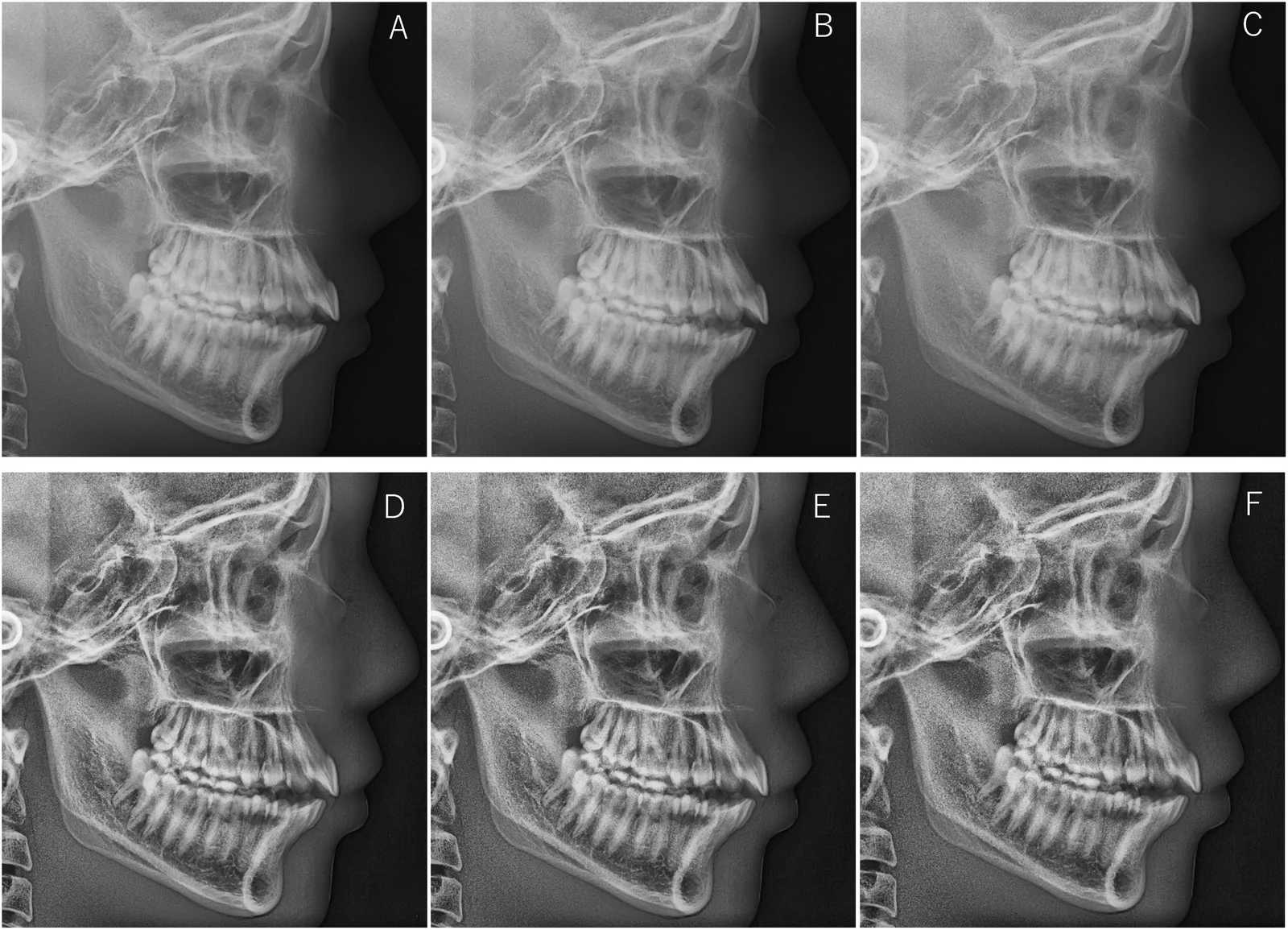
Mildly and markedly sharpened cephalometric radiographs. (A-C) Mildly sharpened cephalometric radiographs (sharpening correction strength: 55%, contrast correction ratio: 20%) acquired at 60%, 40%, and 20% radiation dose, respectively. (D-F) Markedly sharpened cephalometric radiographs (sharpening correction strength: 70%, contrast correction ratio: 70%) acquired at 60%, 40%, and 20% radiation doses, respectively.

### Image visibility evaluation

Six reviewers evaluated the first and second set of cephalometric images First set of 5 images: a 100%-dose image acquired under the clinical routine protocol, a dose-reduced 10%-dose image, and 3 mildly sharpened images acquired at 60%, 40%, and 20% of the routine dose (Figures 1A, E, and 2A-C); second set of 6 images: a 100%-dose image acquired under the clinical routine protocol, a dose-reduced 10%-dose image, a mildly sharpened image at 60% dose, and 3 markedly sharpened images acquired at 60%, 40%, and 20% of the routine dose (Figures 1A, E, and 2A, D-F). The evaluation was conducted using Scheffé’s method of paired comparisons to rank the visibility of the reference point. Each comparative analysis was performed on pairs of lateral cephalometric radiographs arranged side by side. In the first set of 5 images, 10 pairs covered all combinations of images, and in the second set of 6 images, 15 pairs covered all combinations. Reviewers rated the visibility of 5 cephalometric reference points: Nasion (N), the deepest point on the midsagittal section between the anterior nasal spine and the maxillary alveolar margin (Point A), anterior nasal spine (ANS), posterior nasal spine (PNS), and incisal edge of maxillary central incisor (U1), and the visibility of facial soft tissue using a 5-point comparative scale (−2: left image was definitely better; −1: left image was slightly better; 0: images were equal; +1: right image was slightly better; +2: right image was definitely better). The cephalometric images were evaluated twice, once for the first set and once for the second set generated using the 2 sharpening methods described above, with an interval of more than 1 month between the 2 sessions. The 6 reviewers had at least 4 years of experience in assessing the radiographs. One reviewer was a board-certified oral and maxillofacial radiologist, one was a board-certified orthodontist, and the others were trained to become board-certified orthodontists. Before starting, the assessment criteria for the standardized procedure were discussed. All radiographs were reviewed by all reviewers under standardized viewing conditions in a semidarkened room using the same display monitor used for radiographic evaluation (ThinkVision T22i-30, Lenovo Corp., Hong Kong, China). The monitor had a display resolution of 1920 × 1080 pixels and a pixel pitch of 0.247 × 0.247 mm^2^. The brightness and contrast settings were kept constant and standardized for radiographic interpretation throughout the visual assessment. Reviewers were allowed to zoom in and adjust the display magnification as needed.

### Statistical analysis

For the descriptive ranking of conditions, we first summarized the paired-comparison matrix and applied Scheffé’s method of paired comparisons to obtain condition scores and their standard errors, providing an overall ordering of the differences among imaging conditions. For the inferential modeling of reviewer judgments, we specified ordinal logistic regression (proportional-odds models) with a 5-level score as the dependent variable. Reviewer identity was included as a covariate (fixed effect) to adjust for systematic differences in scoring tendency. Results are reported as odds ratios (ORs) relative to the clinical routine image at 100% dose with 95% confidence intervals (CIs); a 2-sided *α* level of 0.05 was used. All ORs were calculated relative to the routine clinical image at 100% dose. An OR>1 indicated superiority over 100%-dose images (ie, a higher probability of better visibility), whereas an OR<1 indicated that the 100%-dose image was superior. Interrater reliability for each reference point was quantified using intraclass correlation coefficients (ICC) with a 2-way random-effects absolute-agreement formulation (ICC (2, *k*) for the mean of *k* = 6 reviewers). As complementary summaries, we report the mean weighted Cohen’s kappa (squared weights) across all rater pairs. EZR software (v1.68; Saitama Medical Center, Jichi Medical University, Saitama, Japan), providing a graphical interface for R (v3.2.2; The R Foundation for Statistical Computing, Vienna, Austria), was used for statistical analyses.

## Results

### Visibility evaluation (first set)

The results of the visibility evaluation for the first set are presented in Table 1. The 10%-dose cephalometric radiograph showed significantly poorer visibility for all 5 reference points and soft tissues than the routine clinical image (100%-dose images). For the mildly sharpened 60%-dose image, the ORs for 4 of the 5 reference points were >1.0, Point A (OR 1.12, *P* = .83), N (OR 2.26, *P* = .11), PNS (OR 2.35, *P* = .1), and U1 (OR 1.35, *P* = .53), indicating a tendency toward equal or better visibility than the 100%-dose image, whereas the ANS had an OR of 0.82; however, none of these differences were statistically significant for any of the reference points. The mildly sharpened 40%-dose image showed a wider spread of ORs across bony landmarks (Point A OR 0.54, *P* = .22; ANS OR 0.61, *P* = .35; N OR 1.84, *P* = .23; PNS OR 1.2, *P* = .7; U1 OR 0.68, *P* = .43), suggesting a possible trend toward equal or better visibility than the 100%-dose image at the N and PNS. Again, no statistically significant differences were observed at any reference point. For the mild-sharpened 20%-dose image, the ORs were <1.0 for 4 of the 5 reference points (Point A OR 0.35, *P = .04; ANS OR 0.28, *P = .017; PNS OR 0.88, *P* = .79; U1 OR 0.47, *P* = .13), except for N (OR 1.14, *P* = .79), indicating a trend toward equal or worse visibility than the 100%-dose image. Among these, ANS showed significantly poorer visibility. In contrast, for all 3 dose levels (60%, 40%, and 20%), the mildly sharpened images demonstrated significantly better soft-tissue visibility than the 100%-dose image.

**Table 1:** Ordinal logistic regression vs clinical image (100% radiation dose): first set.

Reference point	10% dose image OR (95% CI)	*P*-value	60% dose + mild-sharpening image OR (95% CI)	*P*-value	40% dose + mild-sharpening image OR (95% CI)	*P*-value	20% dose + mild-sharpening image OR (95% CI)	*P*-value
Point A	0.054	***	1.12	0.83	0.54	0.22	0.35	*
	(0.015−0.17)	1.9 × 10⁻⁶	(0.41−3.04)		(0.19−1.43)		(0.12−0.94)	0.04
ANS	0.015	***	0.82	0.77	0.61	0.35	0.28	*
	(0.0025−0.064)	2.3 × 10^–7^	(0.29−2.29)		(0.22−1.7)		(0.094−0.78)	0.017
N	0.1	***	2.26	0.11	1.84	0.23	1.14	0.79
	(0.032−0.3)	7.27 × 10^–5^	(0.84−6.28)		(0.68−5.07)		(0.43−3.1)	
PNS	0.066	***	2.35	0.1	1.2	0.7	0.88	0.79
	(0.018−0.21)	9.63 × 10^–6^	(0.85−6.69)		(0.47−3.14)		(0.34−2.27)	
U1	0.055	***	1.35	0.53	0.68	0.43	0.47	0.13
	(0.015−0.18)	3.31 × 10^–6^	(0.53−3.48)		(0.255−1.78)		(0.18−1.24)	
Soft tissue	0.23	**	9.92	***	6.13	**	4.56	**
	(0.072−0.65)	0.0073	(3.26−33.8)	0.0001	(2.17−19.1)	0.001	(1.69−13.2)	0.0036

Proportional-odds ordinal logistic regression with reviewer indicators as covariates. All ORs are calculated relative to the clinical routine image at 100% dose. “Sharpening” denotes the enhancement algorithm applied to low-dose images (60%, 40%, and 20%). The mild-image processing parameters were a sharpening correction strength of 55% and a contrast correction ratio of 20%. An OR > 1 indicates superiority over the 100% dose image; an OR < 1 indicates that the 100% dose image is superior.

*P*-values are Wald tests;

* *P* < .05,

** *P* < .01,

*** *P* < .001.

Abbreviations: 95% CI, 95% confidence interval; ANS, anterior nasal spine; N, Nasion; OR, odds ratio; Point A, the deepest point on the midsagittal section between the anterior nasal spine and maxillary alveolar margin; PNS, posterior nasal spine; U1, incisal edge of the maxillary central incisor.

### Visibility evaluation (second set)

The results of the visibility evaluation for the second set are presented in Table 2. The 10%-dose cephalometric radiograph showed significantly poorer visibility for all 5 reference points compared with the routine clinical image (100%-dose image), and also exhibited poor visibility for the soft-tissue profile. The mildly sharpened 60%-dose image (repeated evaluation of the first set) yielded results that were generally consistent with those obtained in the initial first set evaluation. For markedly sharpened 60%- and 40%-dose image, visibility of all landmarks significantly improved (60%: Point A OR 5.93, ***P = .00024; ANS OR 14.6, ***P = 3.96 × 10^−7^; N OR 3.71, **P = .0047; PNS OR 6.12, ***P = .00014; U1 OR 8.06, ***P = 3.56 × 10^−5^; soft tissue OR 3530, ***P = 1.47 × 10^−9^; 40%: Point A OR 2.64, *P = .04; ANS OR 19.4, ***P = 8.19 × 10^−8^; N OR 7.94, ***P = 3.67 × 10^−5^; PNS OR 5.61, ***P = .00032; U1 OR 7.23, ***P = .00015; soft tissue OR 3410, ***P = 5.49 × 10^−10^). At 20% dose, ANS, N, PNS, U1, and soft tissue remained significantly higher (ANS OR 5.85, ***P = .00043; N OR 3.94, **P = .0044; PNS OR 3.15, *P = .011; U1 OR 4.15, **P = .0035; soft tissue OR 2040, ***P = 1.03 × 10^−9^). At 20% dose, Point A showed a consistent trend toward improvement but not reaching significance (OR 1.19, *P* = .71).

**Table 2:** Ordinal logistic regression vs clinical image (100% radiation dose): second set.

Reference point	10% dose image OR (95% CI)	*P*-value	60% dose + mild-sharpening image OR (95% CI)	*P*-value	60% dose + marked-sharpening image OR (95% CI)	*P*-value	40% dose + marked-sharpening image OR (95% CI)	*P*-value	20% dose + marked-sharpening image OR (95% CI)	*P*-value
Point A	0.12	***	1.41	0.45	5.93	***	2.64	*	1.19	0.71
	(0.04−0.32)	4.1 × 10^⁻5^	(0.58−3.46)		(2.34−15.8)	0.00024	(1.06−6.79)	0.04	(0.48−2.92)	
ANS	0.098	***	0.863	0.74	14.6	***	19.4	***	5.85	***
	(0.0032−0.28)	2.4 × 10^⁻5^	(0.35−2.09)		(5.37−43.2)	3.96 × 10^–7^	(6.84−60.4)	8.19 × 10^–8^	(2.24−16.1)	0.00043
N	0.12	***	1.35	0.52	3.71	**	7.94	***	3.94	**
	(0.043−0.32)	3.2 × 10^⁻5^	(0.54−3.36)		(1.52−9.43)	0.0047	(3.03−21.9)	3.67 × 10^–5^	(1.55−10.3)	0.0044
PNS	0.252	*	2.07	0.11	6.12	***	5.61	***	3.15	*
	(0.1−0.62)	0.011	(0.86−5.06)		(2.45−16)	0.00014	(2.23−14.7)	0.00032	(1.32−7.82)	0.011
U1	0.216	**	1.1	0.84	8.06	***	7.23	***	4.15	**
	(0.081−0.56)	0.0017	(0.45−2.68)		(3.08−22.6)	3.56 × 10^–5^	(2.68−21)	0.00015	(1.62−11)	0.0035
Soft tissue	0.365	0.14	13.2	***	3530	***	3410	***	2040	***
	(0.09−1.37)		(3.5−61)	0.00034	(351−76 000)	1.47 × 10^–9^	(353−64 000)	5.49 × 10^–10^	(237−33 800)	1.03 × 10^–9^

Proportional-odds ordinal logistic regression with reviewer indicators as covariates. All ORs are calculated relative to the clinical routine image at 100% dose. “Sharpening” denotes the enhancement algorithm applied to low-dose images (60%, 40%, and 20%). The mild-image processing parameters were a sharpening correction strength of 55% and a contrast correction ratio of 20%. The marked-image processing parameters were a sharpening correction strength of 70% and a contrast correction ratio of 70%. An OR > 1 indicates superiority over the 100%-dose image; an OR < 1 indicates that the 100%-dose image is superior.

*P*-values are Wald tests;

* *P* < .05,

** *P* < .01,

*** *P* < .001.

Abbreviations: 95% CI, 95% confidence interval; ANS, anterior nasal spine; N, Nasion; OR, odds ratio; Point A, the deepest point on the midsagittal section between the anterior nasal spine and maxillary alveolar margin; PNS, posterior nasal spine; U1, incisal edge of the maxillary central incisor.

### Interrater reliability

The interrater reliabilities of the first and second evaluation image sets are presented in Table 3. Regarding the interrater reliability for the first set, average-measure ICCs (ICC (2,6)) were good reliability (0.819-0.888) for all reference points and soft tissue, indicating that averaging ratings from 6 readers yielded stable assessments. The pairwise weighted Cohen’s kappa showed fair to moderate agreement (median, 0.403-0.538) for all reference points and soft tissues. Regarding the interrater reliability of the second set, the average-measure ICCs (ICC (2,6)) were good to excellent reliability (0.791-0.984), indicating that averaging ratings from 6 readers yielded stable assessments. For ordinal agreement, the pairwise weighted Cohen’s kappa showed fair to almost perfect agreement (median, 0.392-0.898).

**Table 3:** Interrater reliability of the image visibility evaluation.

Reference point	First evaluation image set		Second evaluation image set	
	ICC (2,6)	Weighted Cohen’s kappa	ICC (2,6)	Weighted Cohen’s kappa
Point A	0.82	0.411	0.839	0.463
ANS	0.86	0.505	0.94	0.689
N	0.819	0.403	0.885	0.522
PNS	0.829	0.439	0.791	0.392
U1	0.823	0.493	0.858	0.404
Soft tissue	0.888	0.538	0.984	0.898

Six reviewers; 10 items for the first evaluation, 15 items for the second evaluation.

Abbreviations: ANS, anterior nasal spine; ICC, intraclass correlation coefficient; N, Nasion; Point A, the deepest point on the midsagittal section between the anterior nasal spine and maxillary alveolar margin; PNS, posterior nasal spine; U1, incisal edge of the maxillary central incisor.

## Discussion

The present image visibility analyses (with the routine 100%-dose clinical image as the reference) indicate that post-acquisition image sharpening can largely offset the reduction in landmark visibility caused by dose reduction in cephalometric radiographs. In the first evaluation set, the mild-sharpening images were intentionally tuned to be visually difficult to distinguish from the clinical images, and this “mild” enhancement (sharpening strength 55%, contrast ratio 20%) yielded performance at 60% dose that was overall comparable to, and in several instances numerically better than, the 100%-dose reference. Odds ratios for higher visibility were >1 for Point A, N, PNS, and U1, with no statistically significant difference observed for these landmarks, while soft-tissue visibility was markedly improved (Table 1). Although the 40%-dose mild-sharpening images showed a wider spread of ORs across bony landmarks, none of these differences reached statistical significance, and soft-tissue visibility remained significantly higher than that of the 100%-dose reference (Table 1), collectively suggesting that the 40%-dose approaches equivalence under mild sharpening for several key structures. In contrast, the second evaluation set used a clearly “processed-looking” sharpening setting (marked sharpening; sharpening strength 70%, contrast ratio 70%), and this more aggressive enhancement produced statistically significant superiority over the 100%-dose clinical images not only at 60% dose but also at 40% and 20% dose for most reference points (Table 2). Collectively, these results suggest that recently developed non-AI image-sharpening algorithms can improve the visibility of reference points on low-dose phantom cephalometric radiographs.

To date, radiation dose reduction for conventional dental radiography has largely been achieved through optimization of acquisition-side radioprotective measures, such as beam collimation, additional filtration, selection of the fastest receptor type compatible with the diagnostic task, and circumstantial thyroid shielding, and these strategies have been repeatedly reported to provide meaningful dose savings in the literature.^9–13^ In this study, we demonstrated that applying an image-sharpening software to low-dose cephalometric radiography achieved landmark visibility that was comparable to or better than that obtained with our routine clinical acquisition protocol. These findings suggest that post-acquisition enhancement can compensate for the reduced landmark visibility associated with dose reduction, thereby supporting the feasibility of further investigating this technique as a dose-reduction aid in cephalometric imaging. It has generally been reported that flat panel detector (FPD) systems allow approximately a 50% reduction in radiation dose compared with storage-phosphor systems.^14^^,^^15^ In the present study, using a storage-phosphor system, mild image-sharpening processing enabled at least a reduction to 60% of the conventional dose, suggesting that the use of image sharpening may provide a dose-reduction effect comparable to that achieved by introducing an FPD system, but at substantially lower cost.

A key point of this image-sharpening approach is that it is conceptually different from many conventional “global” post-processing adjustments commonly used in dental radiography (eg, tone-curve/gamma adjustments, histogram-based contrast changes, or simple edge enhancement). The algorithm of the image-sharpening software has been described as enhancing contrast by optimizing the local dynamic range rather than applying a uniform transformation to the entire image, allowing high- and low-contrast regions to be adjusted independently.^6^ In addition, the algorithm aims to restore the image resolution by narrowing the point-spread function, a mechanism explicitly contrasted with typical methods that mainly adjust the contrast dynamic range.^8^ Notably, for low-dose applications, this image-sharpening algorithm is positioned as a rapid, arithmetic enhancement approach that does not rely on AI-based prediction or “drawing in” structures; instead, it emphasizes restoring the inherent image quality without adding content through interpolation/prediction. Collectively, these characteristics provide a rationale for why the present sharpening strategy can preserve (or improve) landmark visibility, even under reduced exposure conditions. The clinical use of this image-sharpening technique has primarily accumulated in ophthalmology, where several studies have reported its application in improving visibility in endoscopic or surgical imaging contexts.^5–8^ To the best of our knowledge, no published studies have validated an image-sharpening approach for dental X-ray imaging. Accordingly, our results position this study as an initial phantom-based technical report supporting further investigation of this specific sharpening technology for dose-reduced cephalometric radiography.

The interrater reliability of the 6-reviewer panel mean achieved good reliability (ICC (2,6): 0.819-0.888) for the first sharpening image set, supporting consensus/averaging in practice. Weighted kappa showed fair to moderate ordinal agreement (0.403-0.538), consistent with frequent near-ties and occasional one-category discrepancies on the 5-point scale. Interrater reliability for the second set showed higher values but was considered indicative only. This is because the second sharpening image set is immediately recognizable, potentially introducing an evaluation bias. Because cephalometric radiographs are interpreted in daily practice by dentists with a wide range of experience, we deliberately assembled a heterogeneous rater panel comprising an oral and maxillofacial radiologist, an orthodontic specialist, and an early career orthodontist with 4 years of clinical experience. Despite this heterogeneity, the interrater agreement remained acceptable, supporting the robustness of the visibility assessment in this phantom study.

This study has several limitations. First, the evaluation of reference point visibility was performed using only adult skull phantom images without the assessment of clinical images obtained from patients. Therefore, the findings cannot be directly extrapolated to pediatric patients, who represent a large proportion of orthodontic patients, because bone density, cortical bone thickness, and craniofacial growth status differ between adults and children. The use of an adult skull phantom was selected for this preliminary study because obtaining pediatric human skull specimens is extremely difficult due to ethical and practical reasons. Second, the outcome measure was limited to subjective visibility of cephalometric reference points and did not directly assess diagnostic accuracy, landmark identification error, or the impact on orthodontic diagnosis and treatment planning. Therefore, the present findings should be interpreted as demonstrating technical feasibility and improved perceived visibility, rather than clinical validity. Future prospective clinical studies using patient images, particularly in pediatric populations, are required to determine whether low-dose lateral cephalometric radiographs enhanced by image-sharpening techniques can maintain diagnostic accuracy in actual clinical practice.

In conclusion, image-sharpening algorithms designed to enhance fine structural detail improve the visibility of reference points on low-dose phantom radiographs. These findings suggest the technical feasibility of image sharpening as a post-acquisition approach for dose-reduced cephalometric imaging.
